# Diagnostic value of the urea-to-creatinine ratio for gastrointestinal bleeding source: influence of renal function

**DOI:** 10.1186/s12882-025-04382-y

**Published:** 2025-08-18

**Authors:** Philipp Russ, Julian M. Koppenhöfer, Simon Bedenbender, Thomas S. Tarawneh, Ulrike W. Denzer, Ivica Grgic, Martin Rußwurm, Christian S. Haas

**Affiliations:** 1 Division of Nephrology, Centre for Internal Medicine, Marburg University, University Hospital Giessen and Marburg, Marburg, Germany; 2 Institute for Artificial Intelligence in Medicine, Marburg University, University Hospital Giessen and Marburg, Marburg, Germany; 3 Division of Hematology and Oncology, Centre for Internal Medicine, Marburg University, University Hospital Giessen and Marburg, Marburg, Germany; 4 Division of Gastroenterology and Endocrinology, Centre for Internal Medicine, Marburg University, University Hospital Giessen and Marburg, Marburg, Germany; 5 Institute of Pharmacology, Marburg University, Marburg, Germany; 6 Division of Nephrology, Centre for Internal Medicine, University Medicine Rostock, Rostock, Germany; 7 Marburg University, Marburg, Germany

**Keywords:** Acute kidney injury (AKI), Chronic kidney disease (CKD), Endoscopy, Gastrointestinal (GI) bleeding, Renal dysfunction, Urea-to-creatinine ratio (UCR)

## Abstract

**Background:**

Gastrointestinal (GI) bleeding is frequent and clinically critical, especially in patients with renal dysfunction. Localization of the source is relevant for treatment and outcomes. The urea-to-creatinine ratio (UCR) has been proposed as a tool to differentiate between upper and lower GI bleeding. However, its diagnostic utility across varying degrees of renal impairment remains unclear.

**Methods:**

From January 2021 to October 2023, all patients with suspected GI bleeding who underwent endoscopy were retrospectively analyzed in a single center. Patients without confirmed bleeding were excluded. Patients were stratified by renal function: normal renal function, chronic kidney disease (CKD), acute kidney injury (AKI), and AKI with pre-existing CKD (acute-on-chronic). We assessed the discriminatory power of the UCR to distinguish upper from lower GI bleeding within each group.

**Results:**

A total of 849 patients (mean age 66.9 ± 18.2 years) were included. Approximately two-thirds of the patients (*n* = 544; 64.1%) were male. CKD was present in 321 (37.8%) and AKI in 354 (41.7%) patients; 199 (56.2%) of those with AKI had pre-existing CKD. Upper GI bleeding occurred more frequently in patients with AKI (75.1%) and CKD (72.9%) than in those with normal renal function (*p* < 0.0001 and *p* = 0.0003, respectively); both groups also required transfusions more frequently (*p* < 0.0001 for both). UCR values were significantly higher in upper vs. lower GI bleeding in patients with normal renal function (67.5 vs. 42.5; *p* < 0.0001), but this difference was reduced in CKD (49.3 vs. 41.0; *p* = 0.0103) and not significant in AKI (53.1 vs. 47.7; *p* = 0.09). The diagnostic performance of UCR was best in patients with normal renal function (AUROC 0.69) and markedly impaired in CKD (AUROC 0.56) and AKI (AUROC 0.54).

**Conclusion:**

The diagnostic value of UCR to localize GI bleeding depends strongly on renal function. While the UCR may help to identify the bleeding site in patients with normal kidney function, impaired renal function hampers the reliability of the UCR, especially in patients with AKI. As renal dysfunction is common in patients with GI bleeding, kidney function must be taken into account when interpreting UCR values.

**Supplementary Information:**

The online version contains supplementary material available at 10.1186/s12882-025-04382-y.

## Introduction

Gastrointestinal (GI) bleeding is a common cause of hospital admissions, accounting for approximately 1–2% of cases and ranking among the top ten causes of urgent GI-related admissions in developed countries [1–3]. Clinical presentation varies depending on the bleeding’s severity and location: upper GI bleeding typically manifests as melena or hematemesis, while lower GI bleeding often presents with hematochezia. However, overlap in symptoms is frequent. Importantly, upper GI bleeding is often more severe and associated with hemodynamic instability. Still, lower GI bleeding can also be life-threatening, particularly in elderly patients with multiple comorbidities [4]. History-taking, physical examination, and assessment of vital signs, as well as laboratory testing form the cornerstone of the diagnostic workup of suspected GI bleeding. Severity and extent of bleeding depend on various factors, including coagulation status, blood count, renal function, and volume status, and determine the necessity for transfusion of blood and coagulation products [5, 6]. In fact, differentiation of upper and lower GI bleeding is clinically relevant since the outcome differs significantly [7]. While endoscopy remains the gold standard for localizing bleeding, it is not always immediately available. As a potential aid, the urea-to-creatinine ratio (UCR) has been proposed, with higher values suggesting upper GI bleeding [8, 9]. This may result from an increased digestion of hemoglobin in the upper GI tract and/or accumulation of urea resulting from renal hypoperfusion [10]. It remains unclear, however, whether UCR is also applicable in patients with impaired kidney function. We therefore aimed to assess the effect of AKI and/or CKD on the UCR and its impact on predicting the localization of GI bleeding. This may be particularly of interest in severe renal hypoperfusion due to GI bleeding, which can in turn lead to prerenal AKI [11, 12]. An increase in UCR might occur independently of the location of the GI bleeding, solely due to increased urea reabsorption in the kidney [13].

The generation of such data is clinically relevant as AKI affects approximately 10–15% of patients admitted to the hospital [14] and CKD is estimated to be present in 11–15% of the global population [15]. The CKD population is a vulnerable group, with higher hospitalization rates and frequent comorbidities such as cardiovascular disease [16]. These patients also face a markedly increased risk of GI bleeding itself, with an estimated incidence of 2% and a twofold increase in mortality. With a mortality rate of 3–7%, GI bleeding is among the leading causes of death in patients with CKD [17]. The availability of a reliable laboratory parameter could prove beneficial in guiding decision-making when the bleeding source cannot be clearly identified based on clinical presentation. We hypothesized that UCR may aid in distinguishing upper from lower GI bleeding, but that its diagnostic value is affected by renal impairment. Therefore, the aim of this study was to evaluate the diagnostic performance of UCR for distinguishing upper from lower GI bleeding, with a specific focus on the influence of renal function.

## Methods

### Patients and study design

**Fig. 1:**
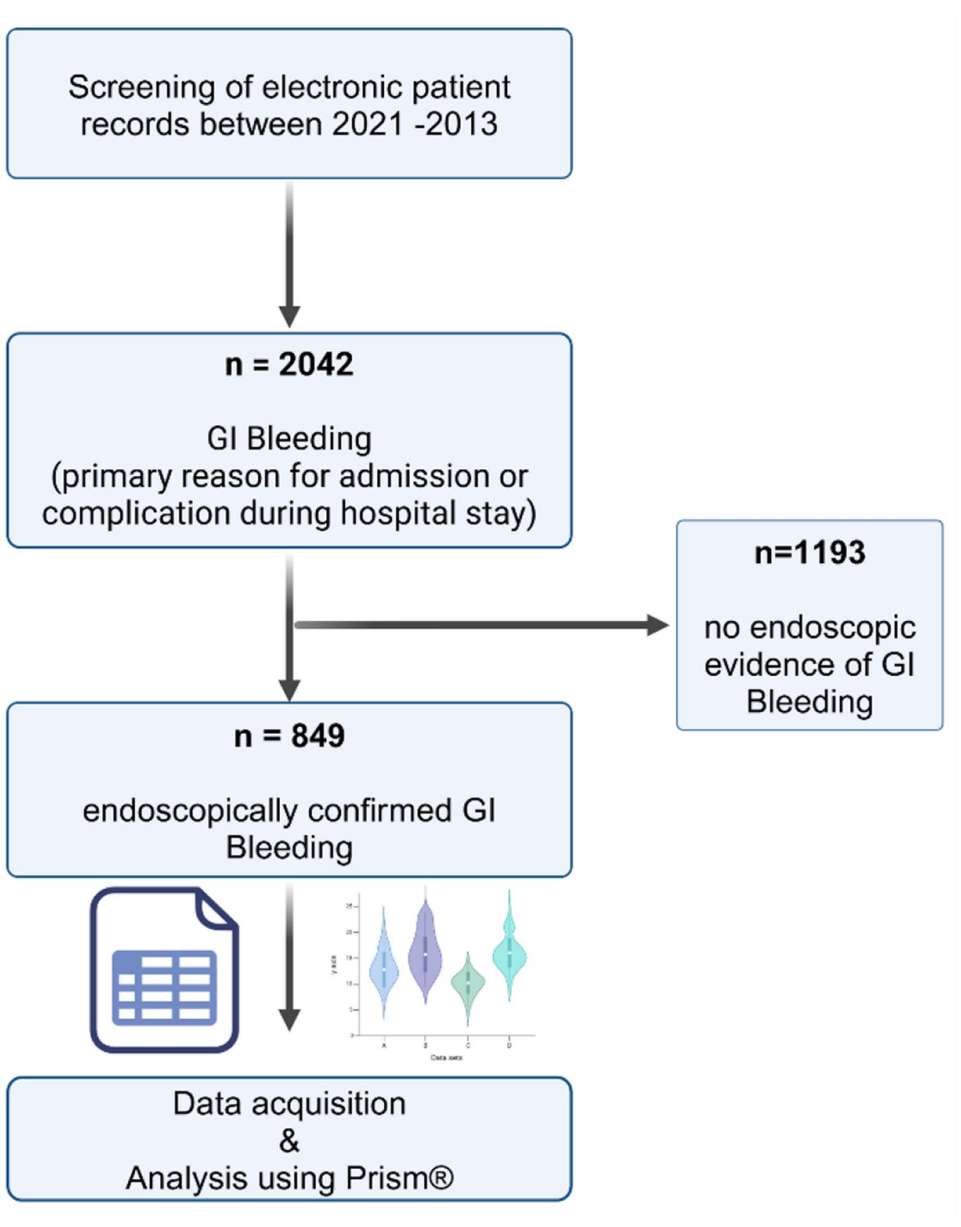
Study design. Gastrointestinal bleeding was confirmed in 849 patients after review of electronic in-patient records and endoscopic findings

In a retrospective, single-center, cross-sectional study, electronic in-patient records of individuals hospitalized between January 2021 and October 2023 at the University Hospital of Marburg, Germany, were systematically searched for the term ‘GI bleeding’. The search included both patients presenting with suspected GI bleeding as a primary diagnosis and those in whom GI bleeding was suspected as a complication during the in-patient stay. Only patients with endoscopic evidence of active or recent GI bleeding (e.g., Forrest Ia, Forrest Ib, Forrest IIa and Forrest IIb ulcers) were included. The study design is summarized in Fig. 1. This research was carried out in accordance with the Declaration of Helsinki of the World Medical Association and was approved by the Ethics Committee of the Philipps University Marburg (file reference ‘23–41 RS’).

### Data acquisition, definition and analysis

Epidemiological data were collected, including patient demographics such as age and sex, along with a detailed account of comorbidities. In addition, endoscopic findings, laboratory parameters, and transfusion requirements were recorded. All laboratory measurements were performed in the same laboratory and all endoscopic procedures were carried out by the same department for all patients.

Differentiation of upper and lower GI tract bleeding was defined by its location relative to the Treitz ligament (i.e., superior duodenal fold): Upper GI bleeding was assumed with bleeding in the esophagus, stomach or duodenum. In contrast, bleeding in the jejunum, ileum, colon and rectum was considered as lower GI bleeding.

Definition of AKI was based upon serum creatinine in accordance with criteria set out in the KDIGO guidelines [18]. AKI stage 1 was defined as an increase in serum creatinine concentration of at least 0.3 mg/dl or a 1.5-fold increase to prior values, while AKI stage 2 was defined as a rise in serum creatinine concentration of at least 2 times the baseline value; AKI stage 3 was defined as present when one of the following criteria were met: a rise in the serum creatinine concentration of at least 3 times the baseline value, an increase of ≥ 4.0 mg/dL, anuria or the initiation of renal replacement therapy. CKD was also defined according to the KDIGO guidelines [19]. A patient was classified as having CKD if the estimated glomerular filtration rate (eGFR) was ≤ 60 mL/min for a period of at least 3 months; in the present study, patients with an eGFR greater than 60 mL/min (CKD1 and CKD2) were considered to have no substantial renal dysfunction. Due to the physiological decline of GFR with aging and the retrospective nature of this study with incomplete urinary data, we focused on an eGFR cutoff of ≤ 60 mL/min to define CKD. Patients with CKD3 were defined as an eGFR between 30 and 59 mL/min, while CKD4 was defined as an eGFR between 15 and 29 mL/min, CKD5 as an eGFR < 15 mL/min and CKD5D as patients requiring permanent dialysis. eGFR was calculated using the MDRD-formula [20]. Patients were classified as having acute on chronic kidney disease if CKD was previously documented or inferred from a persistently reduced eGFR (< 60 mL/min) over at least 3 months prior to the bleeding event, followed by an acute creatinine rise. Classification was performed by the investigators who were blinded to bleeding outcomes. To investigate the effect of renal function on UCR, five groups were analyzed based on the above classification: (1) all patients; (2) patients with normal renal function; (3) patients with CKD3-5D; (4) patients with AKI; and (5) patients with AKI and pre-existing CKD3-5D (acute on chronic).

Serum creatinine and urea were both measured in mg/dL (normal range for creatinine 0.67–1.17 mg/dL and for urea 18–55 mg/dL). The UCR was calculated as the ratio of urea to creatinine.

Data were analyzed using GraphPad Prism^®^ (version 10; Boston, MA, United States). The flow chart was created using BioRender (Toronto, Canada). Results are presented as mean ± standard deviation (SD) and odds ratios (OR) are depicted with 95% confidence intervals (CI). Group differences for dichotomous variables were analyzed using Fisher’s Exact Test. Group differences for continuous variables between characteristics were tested for significance using a two-sample *t*-test. Results were considered statistically significant with a $\:\text{p-value}\le\:0.05$. The effect size was calculated using Cohen’s d. UCR cut-off values of ≥ 75 and ≥ 100 were derived from observed distributions in the dataset, rather than formal optimization methods (e.g., Youden index), as they aligned with clinically relevant clustering in upper GI bleeding cases. Receiver operating characteristic (ROC) curves were generated to assess the diagnostic performance of the UCR in distinguishing upper from lower GI bleeding. The area under the ROC curve (AUROC) with 95% confidence intervals and corresponding p-values were calculated for each subgroup. Statistical comparisons between ROC curves were performed using the DeLong method.

## Results

### Baseline characteristics

**Fig. 2:**
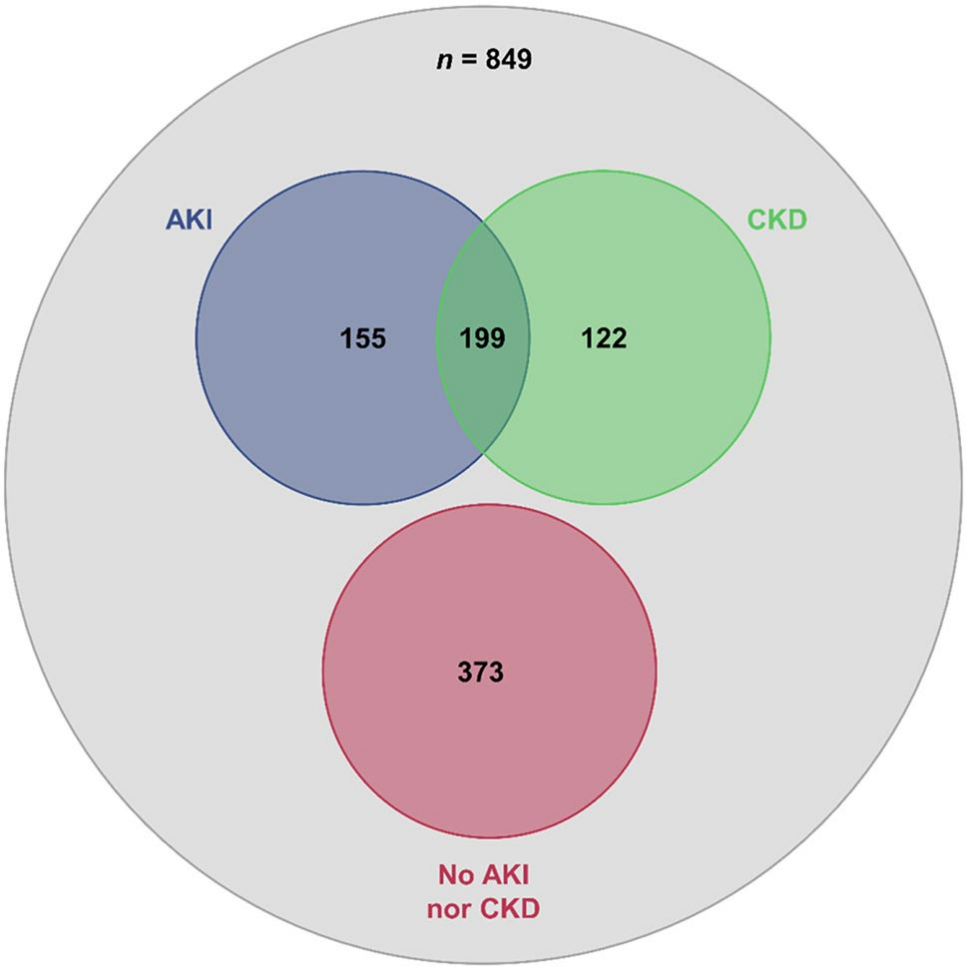
Venn diagram of the study population according to kidney function. Of the 354 patients with AKI, 199 also had CKD. CKD without AKI was present in 122 patients, while 373 patients had normal kidney function (i.e., neither AKI nor CKD)

Overall, 2,042 patients with suspected GI bleeding were identified, of whom 849 patients with endoscopically confirmed GI bleeding were included in the study. The majority were male (554 patients, 65.3%), while 295 patients (34.7%) were female. The average age was 66.9 (± 18.2) years (range 0–99; median 70, IQR 58–81). Upper GI bleeding was observed in 555 patients (65.4%), while 294 patients (34.6%) had lower GI bleeding. The most common endoscopic bleeding diagnoses were Forrest ulcer bleeding (26.6%), angiodysplasias (10.7%), post-interventional bleeding (7.9%), tumors (7.4%), and diverticular bleeding (6.9%). AKI was present in 354 patients (41.7%), of whom 56.2% (*n* = 199) had pre-existing CKD. Of the remaining study population, 321 patients (37.8%) had CKD, while 373 patients (43.9%) had normal renal function, i.e., neither AKI nor CKD (Table 1, Supplemental Table 1 and Fig. 2).

**Table 1:** Baseline characteristics, entire cohort

***Sex – no.***		
Female	295	34.7%
Male	554	65.3%
***Age – years***		
Mean ± standard deviation	66.9 ± 18.2	-
Min < median < max	0 < 70 < 99	-
***Localization of GI bleeding – no.***		
Upper GI bleeding	555	65.4%
Lower GI bleeding	294	34.6%
***Kidney disease/injury – no.***		
* Acute kidney injury (AKI)*	354	41.7%
AKI Stage 1	219	25.8%
AKI Stage 2	57	6.7%
AKI Stage 3	78	9.2%
Acute on chronic kidney injury	199	23.4%
* Chronic kidney disease (CKD) 3-5D*	321	37.8%
CKD3	201	23.7%
CKD4	74	8.7%
CKD5 and CKD5D	46	5.4%
***Other concomitant diseases – no.***		
Diabetes mellitus	194	22.9%
Hypertension	531	62.5%
Coronary artery disease	199	23.4%
Peripheral arterial occlusive disease	94	11.1%
Stroke	103	12.9%
Atrial fibrillation	167	19.7%
***Premedication – no.***		
Platelet aggregation inhibitor	286	33.7%
New oral anticoagulants, heparin or phenprocoumon	244	28.7%
***Transfusion requirement per hospitalization admission – no.***		
Patients transfused with red blood cell concentrates	456	53.7%

GI, gastrointestinal

Overview of demographic data, localization of Gastrointestinal bleeding, kidney function status, relevant comorbidities, pre-existing anticoagulant or antiplatelet therapy, and transfusion requirements among 849 patients with endoscopically confirmed GI bleeding

Patients with CKD were significantly more likely to have hypertension (*p* < 0.0001; OR 2.41, CI 1.78 to 3.26), diabetes (*p* < 0.0001; OR 2.68, CI 1.92 to 3.71), antiplatelet therapy (*p* = 0.0021; OR 1.59, CI 1.19 to 2.11), and oral anticoagulants (*p* < 0.0001; OR 2.09, CI 1.56 to 2.82). Moreover, CKD patients presented with a significantly lower hemoglobin level on admission than patients with normal kidney function (95.6 ± 28.3 g/L vs. 111.3 ± 74.0 g/L, *p* = 0.0003; Table 2).

**Table 2:** Concomitant diseases and premedication with regard to kidney function

Kidney function	Value	Proportion of subgroup
**Patients with normal kidney function**	**373**	
Upper GI bleeding	207	55.5%
Lower GI bleeding	166	44.5%
Diabetes mellitus	69	18.5%
Hypertension	209	56.0%
Coronary artery disease	73	19.6%
Peripheral arterial occlusive disease	25	6.7%
Stroke	30	8.0%
Atrial fibrillation	48	12.9%
Platelet aggregation inhibitor	116	31.1%
New oral anticoagulants, heparin or phenprocoumon	70	18.8%
Hemoglobin at the time of admission (mean)	111.7 g/L	---
Hemoglobin at the time of endoscopy (mean)	98.8 g/L	---
Hemoglobin lowest (mean)	89.4 g/L	---
Patients transfused with RBC concentrates	151	40.5%
Total number of RBC concentrates transfused	580	---
***Chronic kidney disease***	***321***	
Upper GI bleeding	234	72.9%
Lower GI bleeding	87	27.1%
Diabetes mellitus	109	34.0%
Hypertension	240	74.8%
Coronary artery disease	101	31.5%
Peripheral arterial occlusive disease	53	16.5%
Stroke	56	17.4%
Atrial fibrillation	102	31.8%
Platelet aggregation inhibitor	129	40.2%
New oral anticoagulants, heparin or phenprocoumon	123	38.3%
Hemoglobin at the time of admission (mean)	95.6 g/L	---
Hemoglobin at the time of endoscopy (mean)	86.6 g/L	---
Hemoglobin lowest (mean)	74.5 g/L	---
Patients transfused with RBC concentrates	205	63.9%
Total number of RBC concentrates transfused	1098	---
***Acute kidney injury (including AKI with preexisting CKD)***	***354***	
Upper GI bleeding	266	75.1%
Lower GI bleeding	88	24.9%
Diabetes mellitus	96	27.1%
Hypertension	228	64.4%
Coronary artery disease	84	23.7%
Peripheral arterial occlusive disease	43	12.1%
Stroke	55	15.5%
Atrial fibrillation	97	27.4%
Platelet aggregation inhibitor	111	31.4%
New oral anticoagulants, heparin or phenprocoumon	124	35.0%
Hemoglobin at the time of admission (mean)	102.5 g/L	---
Hemoglobin at the time of endoscopy (mean)	90.6 g/L	---
Hemoglobin lowest (mean)	77.0 g/L	---
Patients transfused with RBC concentrates	234	66.1%
Total number of RBC concentrates transfused	1462	---

GI, gastrointestinal; RBC, red blood cell

Subgroup analysis comparing GI bleeding localization, comorbidities, medication use, hemoglobin values, and transfusion needs among patients with normal renal function, CKD, or AKI

**Fig. 3:**
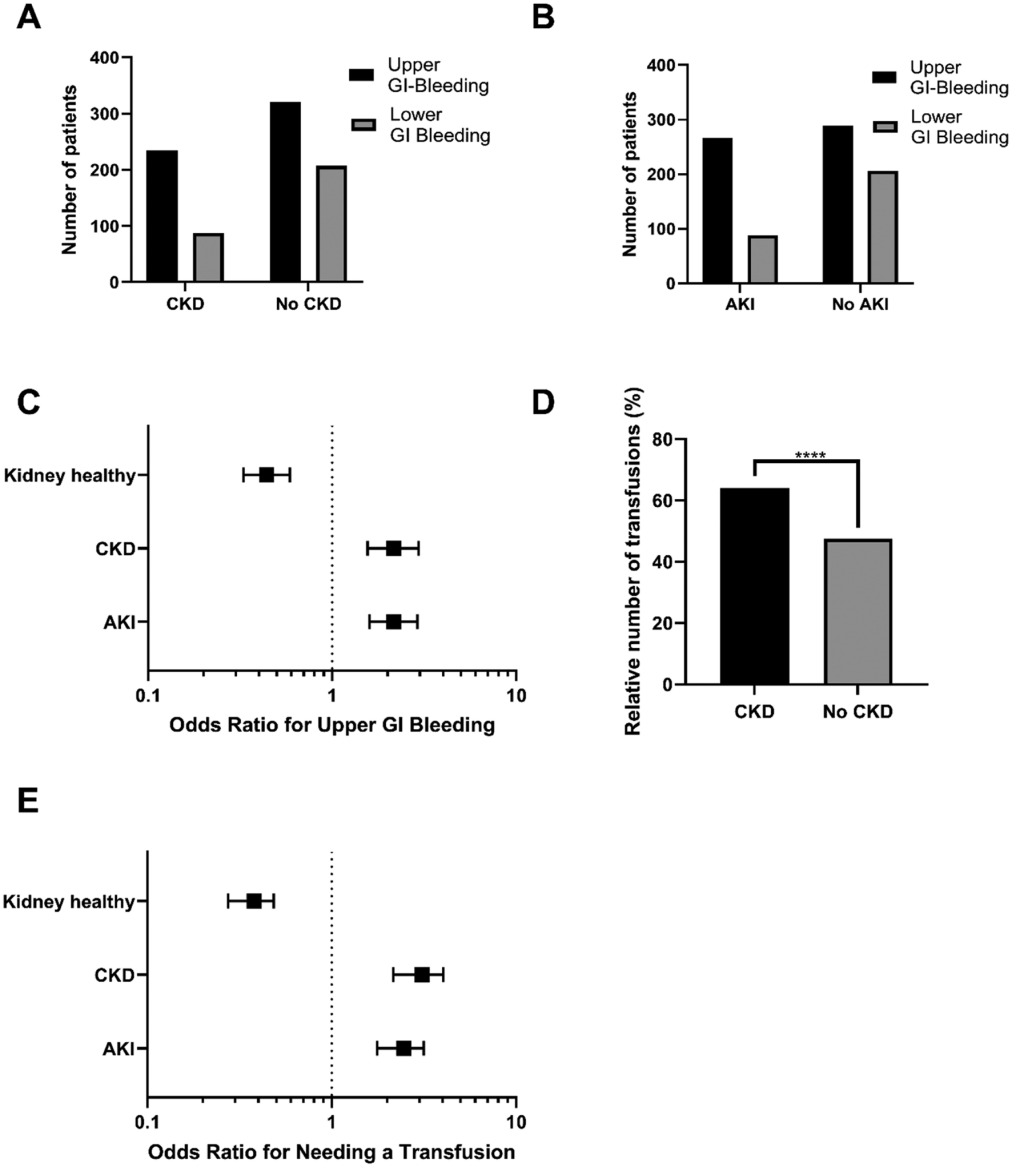
Occurrence of site of GI bleeding and transfusion in relation to renal impairment. (**A** and **B**) Localization of bleeding in patients with CKD (**A**) and AKI (**B**). Upper GI bleeding is more common in patients with CKD and AKI (*p* = 0.0003 and *p* < 0.0001, respectively). (**C**) Risk of upper GI bleeding. Both patients with AKI and patients with CKD have an increased risk of upper GI bleeding compared to those with normal kidney function (OR 2.16; CI 1.56 to 2.95 and OR 2.16; CI 1.60 to 2.91, respectively). (**D**) Transfusion requirement. Patients with CKD were significantly more likely to require transfusions (63.9% vs. 47.5%; **** = *p* < 0.0001). (**E**) Probability of transfusion according to renal involvement. Both CKD and AKI patients are more likely to require transfusion during their hospital stay if they presented with GI bleeding (OR 3.00; CI 2.21 to 4.08 and OR 2.40; CI 1.80 to 3.19, respectively)

Upper GI bleeding occurred more frequently in patients with CKD (72.9%) than in those without CKD (60.8%; *p* = 0.0003, Fig. 3A). Furthermore, AKI was significantly more frequently associated with upper GI bleeding compared to patients without AKI (75.1% vs. 58.4%, respectively; *p* < 0.0001; Fig. 3B). Compared to patients without renal disease (neither AKI nor CKD), patients with CKD (OR 2.16; CI 1.56 to 2.95) and AKI (OR 2.16; CI 1.60 to 2.91) presented more often with upper GI bleeding (Fig. 3C).

### AKI and CKD patients with GI bleeding require more transfusions

Patients with CKD and GI bleeding had a higher likelihood of requiring transfusion during hospitalization than those without CKD, irrespective of concomitant AKI (63.9% vs. 47.5%; *p* < 0.0001; Fig. 3D). Similarly, patients with AKI were significantly more likely to require transfusions than patients without AKI (*p* < 0.0001). Compared to patients with normal renal function, both patients with CKD and those with AKI were more likely to require transfusion in case of GI bleeding (OR 0.37, CI 0.28 to 0.49 vs. OR 3.00, CI 2.21 to 4.08 and OR 2.40, CI 1.80 to 3.19, respectively; Fig. 3E). In terms of transfusion requirements, patients without kidney disease received 3.8 (± 3.2) red blood cell (RBC) units on average, while patients with CKD or AKI needed more transfusions during their hospital stay (5.3 ± 5.4; 6.2 ± 6.3 RBCs).

### Diagnostic performance of the UCR according to renal function

**Fig. 4:**
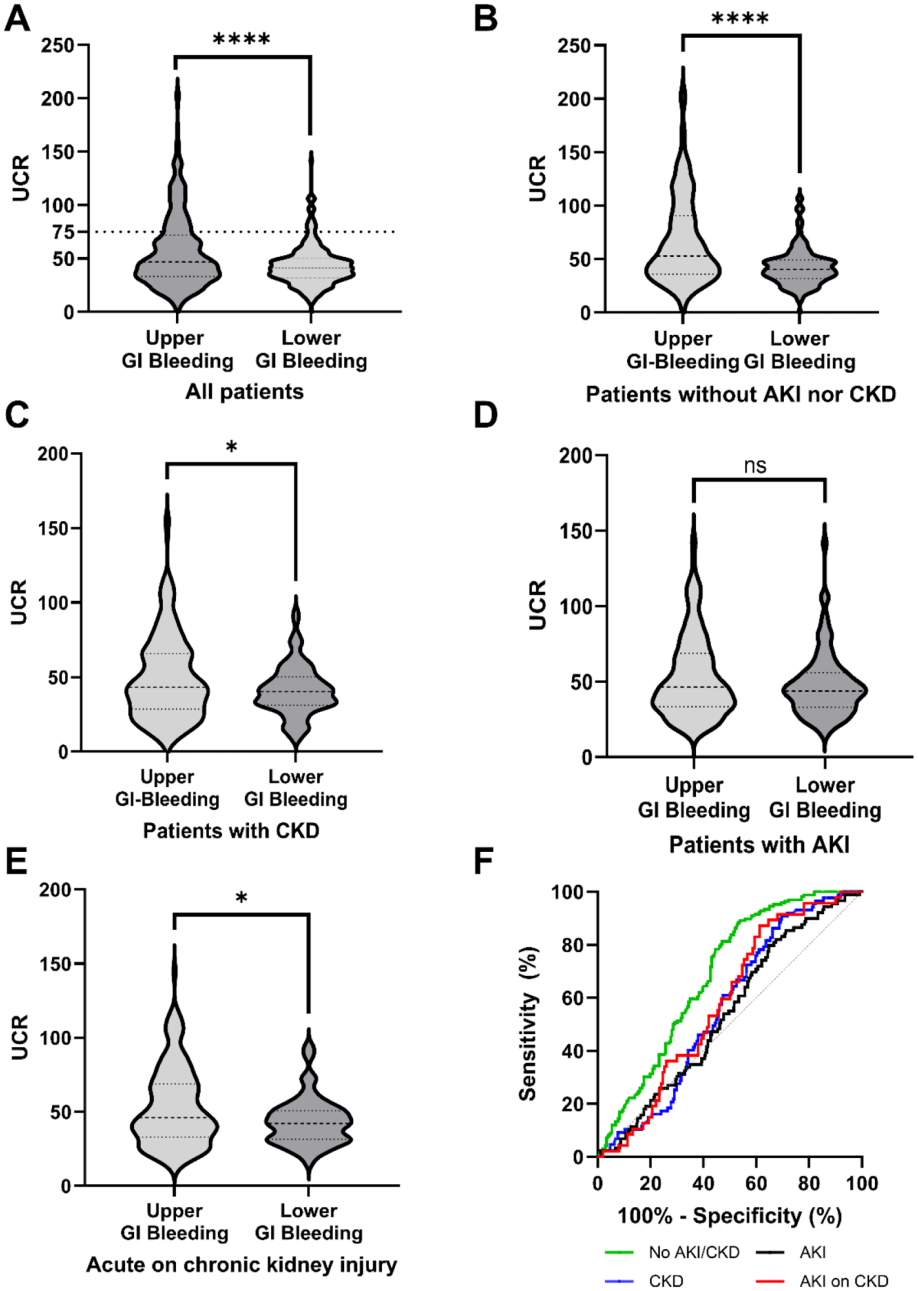
UCR for differentiation of upper and lower GI bleeding according to renal function. (**A**) UCR in the overall cohort. Upper GI bleeding showed a significantly higher UCR (**** = *p* < 0.0001). A UCR (red threshold line) above 75 or 100 was highly sensitive for upper GI bleeding (93.5% and 98.3%), but poorly specific (23.8% and 12.4%). (**B**) UCR in patients without CKD or AKI. The UCR for upper GI bleeding was also significantly higher in patients without AKI and CKD (**** = *p* < 0.0001). (**C**) UCR in patients with CKD. In patients with CKD, there was only slightly but significantly higher in UCR for upper GI bleeding (* = *p* < 0.05). (**D**) UCR in patients with AKI. There were no significant differences in UCR between upper and lower GI bleeding in patients with AKI (ns, *p* = 0.09). (**E**) UCR in patients with acute on chronic kidney injury. In patients with acute on chronic kidney injury, there was a weakly significant increase in the UCR. (**F**) Receiver operating characteristics (ROCs) curves for all subgroups. The largest areas under the ROC curves (AUROCs) were found in those without CKD and AKI, whereas the AUROCs were lower in those with AKI (0.69 vs. 0.54; *p* < 0.0001). Thick dashed line represents median, thin dashed lines represent the 25th and 75th percentiles, respectively

In the overall cohort, UCR was significantly higher in patients with upper GI bleeding than in those with lower GI bleeding (56.8 ± 34.2 vs. 43.4 ± 18.7; *p* < 0.0001); however, this was only a moderate effect (Cohen’s d = 0.45). In the overall cohort, a UCR threshold of ≥ 75 yielded a sensitivity of 93.5% (CI 90.1–95.8) and a specificity of 23.8% (CI 20.3–27.3) for detecting upper GI bleeding. Increasing the threshold to ≥ 100 raised sensitivity to 98.3% (CI 96.1–99.3), while specificity dropped further to 12.4% (CI 9.9–15.4). These data indicate that while high UCR values are commonly found in upper GI bleeding, the lack of specificity limits their utility in distinguishing bleeding sites (Fig. 4A).

In patients without kidney disease (neither AKI nor CKD), the UCR was even higher in patients with upper than in lower GI bleeding (67.5 ± 40.5 vs. 42.5 ± 17.3; *p* < 0.0001), representing a large effect (Cohen’s d = 0.77; Fig. 4B).

In contrast, patients with CKD showed only a marginally elevated UCR for upper GI bleeding when compared to CKD patients with lower GI bleeding (49.3 ± 27.9 vs. 41.0 ± 16.6; *p* = 0.0103; Cohen’s d = 0.32; Fig. 4C).

In patients with AKI, there was no significant difference in the UCR (53.1 ± 27.0 vs. 47.7 ± 21.2; *p* = 0.09; Cohen’s d = 0.22, Fig. 4D). However, there was a moderate but significant difference in the UCR in patients with AKI and pre-existing CKD (53.0 ± 26.1 vs. 43.7 ± 15.9; *p* < 0.05; Cohen’s d = 0.38; Fig. 4E).

ROC analysis demonstrated that the diagnostic performance of the UCR in predicting upper GI bleeding varied significantly depending on renal function. Among patients with normal renal function (i.e., neither AKI nor CKD), the area under the ROC curve (AUROC) was 0.69 (95% CI 0.63–0.74; *p* < 0.0001). In the overall cohort, irrespective of renal status, the AUROC was 0.60 (95% CI 0.56–0.64; *p* < 0.0001). In contrast, patients with CKD had a lower AUROC of 0.56 (95% CI 0.50–0.63; *p* = 0.08), while those with acute-on-chronic kidney disease had an AUROC of 0.58 (95% CI 0.50–0.67; *p* = 0.09). The lowest AUROC was observed in patients with AKI alone, with a value of 0.54 (95% CI 0.48–0.60; *p* = 0.26), indicating no significant discriminatory value in this subgroup.

A statistical comparison of ROC curves using the DeLong test revealed significant differences between patients with normal renal function and those with AKI (0.69 vs. 0.54; *p* < 0.0001), as well as between those with normal renal function and those with CKD (0.69 vs. 0.56; *p* < 0.0001). However, there was no statistically significant difference between the AUROC curves between patients with AKI and those with CKD (0.54 vs. 0.56; *p* = 0.67) (Fig. 4F).

## Discussion

Our study confirms previous findings that a high UCR may be useful in differentiating upper from lower GI bleeding [9]. However, earlier studies often excluded patients with impaired renal function when assessing the diagnostic accuracy and utility of UCR [8, 21]. For instance, Ernst et al. reported an AUROC of 0.73 in their overall cohort [8], which is comparable to the AUROC of 0.69 we observed in patients with normal renal function. Importantly, our study demonstrates that patients with AKI have a significantly lower AUROC of 0.54 (*p* < 0.0001), a factor not considered in prior research. Given that a substantial proportion of hospitalized patients present with impaired kidney function [22–24], with over 40% of the present cohort diagnosed with AKI, our study filled this gap by systematically evaluating UCR performance across different renal function subgroups. Our findings highlight that while UCR retains some discriminatory value in patients with CKD, its diagnostic accuracy is significantly reduced in those with AKI.

### Reduced diagnostic performance of UCR in renal impairment

The limitation in patients presenting with AKI may partly result from the fact that AKI associated with GI bleeding is often of prerenal origin [11, 12], reflecting a potentially bidirectional relationship between GI bleeding and renal impairment. GI bleeding causes renal hypoperfusion, resulting in increased urea reabsorption in the proximal tubule [25]. Reduced renal perfusion leads to increased reabsorption of water and sodium due to activation of the renin-angiotensin-aldosterone system, which is passively accompanied by urea due to the resulting increased concentration gradient. Additionally, more urea transporters (UT-A1) are incorporated into the luminal membrane of the collecting duct mediated by antidiuretic hormone [26], which may contribute to a greater reduction in urea clearance than creatinine clearance. Consistently, UCR is already elevated in prerenal AKI [13, 27] and not only altered by GI bleeding, which may explain the lack of GI bleeding effect in this population. As mentioned, this was supported by ROC analysis, which showed a significantly higher AUROC for patients with normal renal function compared to those with either AKI or CKD (*p* < 0.0001 for both). In contrast, there was no significant difference between the AKI and CKD groups (*p* = 0.67). This reduced discriminatory power appears to result from elevated UCR values even in patients with lower GI bleeding and AKI, thereby diminishing the contrast between bleeding sources.

This interpretation is further supported by diagnostic performance metrics. In the overall cohort, a UCR threshold of ≥ 75 resulted in a high sensitivity of 93.5%, but a low specificity of only 23.8%. Raising the threshold to ≥ 100 increased sensitivity to 98.3%, while specificity dropped to just 12.4%. These findings highlight that although elevated UCR values are common in upper GI bleeding, they also occur in lower GI bleeding, particularly in patients with renal dysfunction. This limits the discriminatory value of UCR in unselected clinical populations. Moreover, as all patients in our study had confirmed GI bleeding, these metrics reflect the ability of UCR to differentiate the source of bleeding rather than detect bleeding itself. The lack of a healthy control group prevents calculation of predictive values. Nevertheless, the findings remain highly relevant for real-world hospital settings, where the clinical question often centers not on whether bleeding is present, but where it originates.

### Potential role of UCR in patients presenting with CKD

Our data demonstrate a significantly lower UCR in patients with CKD compared to those with normal renal function. This indicates a disproportionately greater increase in creatinine compared to urea. Despite the lower UCR, there is still a significant difference between CKD patients with upper and lower GI bleeding. UCR may be a useful parameter to identify the localization of GI tract bleeding not only in the overall cohort but also in patients with CKD, affecting approximately 11–15% of the global population [15]. Thus, despite the limited reliability of UCR, it could be used in CKD patients to guide the next diagnostic step in resource-limited settings where immediate endoscopy is less available. In fact, UCR may further expedite identification of the bleeding source and guide the decision whether to perform esophagogastroduodenoscopy or colonoscopy, ultimately enhancing patient care and outcomes. However, as mentioned, UCR appears to be of limited utility in AKI or if renal function is not taken into account. Notably, prior creatinine values are often not immediately available or considered in cases of acute GI bleeding, and unfortunately render the UCR unsuitable in most circumstances.

### Association between renal disease and bleeding risk

Our data show that upper GI bleeding is more common in patients with CKD, consistent with the prevailing literature suggesting that patients with CKD have an increased risk of bleeding [17, 28, 29]. A possible cause of the increased risk of bleeding may be platelet dysfunction in patients with kidney disease [30–32]. In addition, patients with CKD suffer from multiple comorbidities requiring anticoagulation or antithrombotic therapy. Moreover, some of these comorbidities inherently increase the risk of bleeding [33]. For example, diabetes, as the most common cause of CKD [34], has been shown to increase the risk of GI bleeding [35]. Our data are consistent with the literature, showing that CKD patients are significantly more likely to have hypertension, diabetes, antiplatelet and anticoagulation therapy than patients without known CKD. This suggests that the study population is representative.

### Increased transfusion needs in renal disease

We also observed that patients with CKD or AKI and GI bleeding need transfusions more often than patients with normal kidney function. Iron deficiency and renal anemia due to erythropoietin deficiency may increase transfusion needs and reduce regenerative capacity [36]. Consistently, CKD patients in this study had lower hemoglobin levels on admission. Another reason may be a lower transfusion threshold due to comorbidities. For example, patients with CKD are more likely to have cardiovascular disease [37], which, depending on the guidelines, may ultimately result in earlier transfusions [38, 39].

### Limitations

There are some limitations to the study. First, the study design was retrospective and conducted in a single center. Due to the retrospective design, our study cannot establish causality between GI bleeding and AKI; moreover, the potentially bidirectional relationship between these conditions complicates the interpretation of UCR values. Furthermore, as patients were enrolled in a university hospital, they may be more critically ill and the results may not be fully generalizable to other clinical settings. Second, only patients with proven GI bleeding were included, and no comparison with healthy subjects was made. Additionally, the UCR may be influenced by other factors, such as diet or exercise status, and preexisting medications, such as diuretics, which were not considered. Finally, statistical significance cannot automatically be translated into clinical benefit, and it remains uncertain whether the rather small effect sizes are sufficient to justify clinical application.

## Conclusion

Our data suggest that: (1) upper GI bleeding is more common in patients with either AKI or CKD; (2) a significant proportion of patients with GI bleeding present with concomitant AKI; (3) patients with CKD or AKI more frequently required transfusions; and (4) while a high UCR may help distinguish between upper and lower GI bleeding, its diagnostic value is significantly limited in patients with impaired renal function, particularly those with AKI.

To our knowledge, this is one of the first studies to systematically evaluate the performance of UCR across varying degrees of kidney function. These findings underscore that UCR should not be interpreted in isolation but rather in the context of renal parameters. Therefore, UCR may help guide the decision for targeted endoscopy in selected patients, provided that renal function is taken into account.

## Supplementary Information

Below is the link to the electronic supplementary material.

Supplementary Material 1

## Data Availability

All relevant data is reported in the article. Additional data can be provided by the corresponding author on reasonable request.

## Abbreviations

AKI: Acute kidney injury
AUROC: Area under receiver operating characteristics
CI: Confidence interval
CKD: Chronic kidney disease
GI: Gastrointestinal
OR: Odds ratio
ROC: Receiver operating characteristics
UCR: Urea-to-creatinine ratio
