# Supplementary material for: Diagnostic value of the urea-to-creatinine ratio for gastrointestinal bleeding source: influence of renal function
Source: BMC Nephrol. 2025 Aug 18;26:464. doi: 10.1186/s12882-025-04382-y (PMC12359887; doi:10.1186/s12882-025-04382-y)
Supplement: Supplementary file 1 — Supplementary Material 1 [file 12882_2025_4382_MOESM1_ESM.docx]

***Supplementary Material***

***Diagnostic Value of the Urea-to-Creatinine Ratio for Gastrointestinal Bleeding Source: Influence of Renal Function***

Supplementary Table 1 | Endoscopic findings of the entire cohort. Some patients had multiple findings during a single endoscopic examination.

| ***Endoscopic finding – no.*** | | |
| --- | --- | --- |
| Forrest bleeding ulcers | 226 | 26.6% |
| Angiodysplasias | 91 | 10.7% |
| Post-resection/intervention bleeding | 67 | 7.9% |
| Tumor bleeding | 63 | 7.4% |
| Diverticular bleeding | 59 | 6.9% |
| Bleeding due to reflux esophagitis | 53 | 6.2% |
| Bleeding in inflammatory bowel disease (IBD) | 44 | 5.2% |
| Esophageal varices | 42 | 4.9% |
| Ulcer of colon/rectum | 23 | 2.7% |
| Mucosal bleeding, unspecified | 23 | 2.7% |
| Iatrogenic mechanical bleeding | 21 | 2.5% |
| Anastomotic bleeding | 17 | 2.0% |
| Bleeding in proctitis/colitis | 16 | 1.9% |
| Papillotomy/papillary bleeding | 15 | 1.8% |
| Polyp bleeding/adenoma | 14 | 1.6% |
| Bleeding in ischemic colitis | 14 | 1.6% |
| Mallory-Weiss lesion | 13 | 1.5% |
| Erosive/hemorrhagic gastritis/duodenitis | 12 | 1.4% |
| Fundic varices | 10 | 1.2% |
| Pressure ulceration | 8 | 0.9% |
| Bleeding due to portal hypertensive gastropathy | 8 | 0.9% |
| Post argon plasma coagulation bleeding | 6 | 0.7% |
| Osler’s disease (Hereditary hemorrhagic telangiectasia) | 5 | 0.6% |
| Diffuse gastric mucosal bleeding | 5 | 0.6% |
| Bleeding from axial hernia | 5 | 0.6% |
| Bleeding due to small bowel ischemia | 4 | 0.5% |
| Bleeding in gastroenteritis | 4 | 0.5% |
| Bleeding due to perforation | 4 | 0.5% |
| Iatrogenic drug-induced bleeding | 3 | 0.4% |
| Haemobilia | 2 | 0.2% |
| Hemorrhoids | 2 | 0.2% |
| Bleeding from glandular cyst | 2 | 0.2% |
| Diffuse hemorrhagic esophagitis | 1 | 0.1% |
| Bleeding in necrotizing esophagitis | 1 | 0.1% |
| Bleeding in Henoch-Schönlein Purpura | 1 | 0.1% |
| Unspecified or undiagnosed and other bleeding sources | 45 | 5.3% |
